# Successful Direct Current Cardioversion (DCCV) in Pregnancy in a Non-Obstetric Emergency Department

**DOI:** 10.7759/cureus.73419

**Published:** 2024-11-11

**Authors:** Haleeda Hilmi, Leah Flanagan, Eamonn Murphy, Bibi Bassa, Cian McDermott

**Affiliations:** 1 Emergency Medicine, Mater Misericordiae University Hospital, Dublin, IRL; 2 Cardiology, Mater Misericordiae University Hospital, Dublin, IRL; 3 Trauma Inpatient Service, Mater Misericordiae University Hospital, Dublin, IRL

**Keywords:** arrhythmia management, atrial fibrillation, cardioversion, direct current cardioversion, pregnancy

## Abstract

Atrial Fibrillation (AF) is uncommon in pregnancy but associated with significant mortality. Although controlled studies evaluating therapeutic management of AF in pregnancy are lacking, current guidelines suggest that direct current cardioversion (DCCV) is safe in cases of maternal arrhythmia with hemodynamic compromise. In this report, we discuss a female patient of 22 weeks gestation who presented to the non-obstetric Emergency Department (ED) with acute onset, symptomatic AF. Following consultation with emergency medicine, cardiology, and maternal-fetal medicine specialists, rhythm control was favored for immediate management. A single 200 joules synchronized shock resulted in a successful reversion to sinus rhythm with no adverse events using agreed procedural sedation protocols. The patient had an uneventful inpatient course and was later discharged with outpatient cardiology and obstetric follow-up. This case contributes to the evidence that DCCV is safe in pregnant patients and proposes that standard medications used for analgesia and sedation in cardioversion are safe in pregnancy. A multidisciplinary team approach is key in managing AF in pregnancy in the acute non-obstetric setting.

## Introduction

Atrial fibrillation (AF) is relatively uncommon in pregnancy [1]. It is one of the most common forms of arrhythmia and is associated with increased maternal and fetal morbidity and mortality [2-4]. The rapid ventricular response may have detrimental hemodynamic consequences for both mother and fetus [5,6].

There is limited availability of high-quality controlled studies evaluating the management of atrial fibrillation in pregnancy. Concerns regarding teratogenicity and adverse fetal side effects have been raised with the use of antiarrhythmic drugs (AADs), especially in the first trimester of pregnancy [7]. The Food and Drug Administration (FDA) made available a five-letter classification system of potential AADs that can cause harm in pregnancy in 1979. These classifications have been revised by several publications in favor of a more native assessment of risk, including notable agents such as sodium channel blockers, calcium channel blockers, and beta blockers. [8]. There is a significant paucity of randomized controlled trials (RCTs) about the use of AADs, and thus, it is recommended that we continue to practice a risk vs. benefits approach to their use and use the lowest, effective dose [1,7,8].

Current international consensus supports the use of direct current cardioversion (DCCV) in pregnant patients with hemodynamic instability [9]. A recently published confidential inquiry into maternal deaths in the United Kingdom indicated inadequate management of underlying arrhythmias during pregnancy when compared to the standard of care received by their non-pregnant control [10,11]. This case report demonstrates the safe and successful use of DCCV under the stewardship of a multidisciplinary team in the management of AF in a stable pregnant patient in the non-obstetric ED.

## Case presentation

A 43-year-old female presented to the ED of a non-obstetric academic urban hospital with acute onset palpitations and light-headedness lasting three hours. The patient had been well prior to the onset of symptoms and reported no obvious precipitants, medications, recreational drugs, or alcohol use. 

On further inquiry, the patient was 22 weeks pregnant following in vitro fertilization (IVF). She had one uncomplicated pregnancy carried to term and a history of previous miscarriages. The patient was under the care of local fetal-maternal medicine specialists and reported no hematological disorders. The patient had no other past medical history and no diagnosed hypertension or diabetes perinatally, although she has reported prior episodes of palpitations with the normal investigation. Over the course of index pregnancy, the patient did note an increase in the frequency of palpitations. 

**Figure 1:**
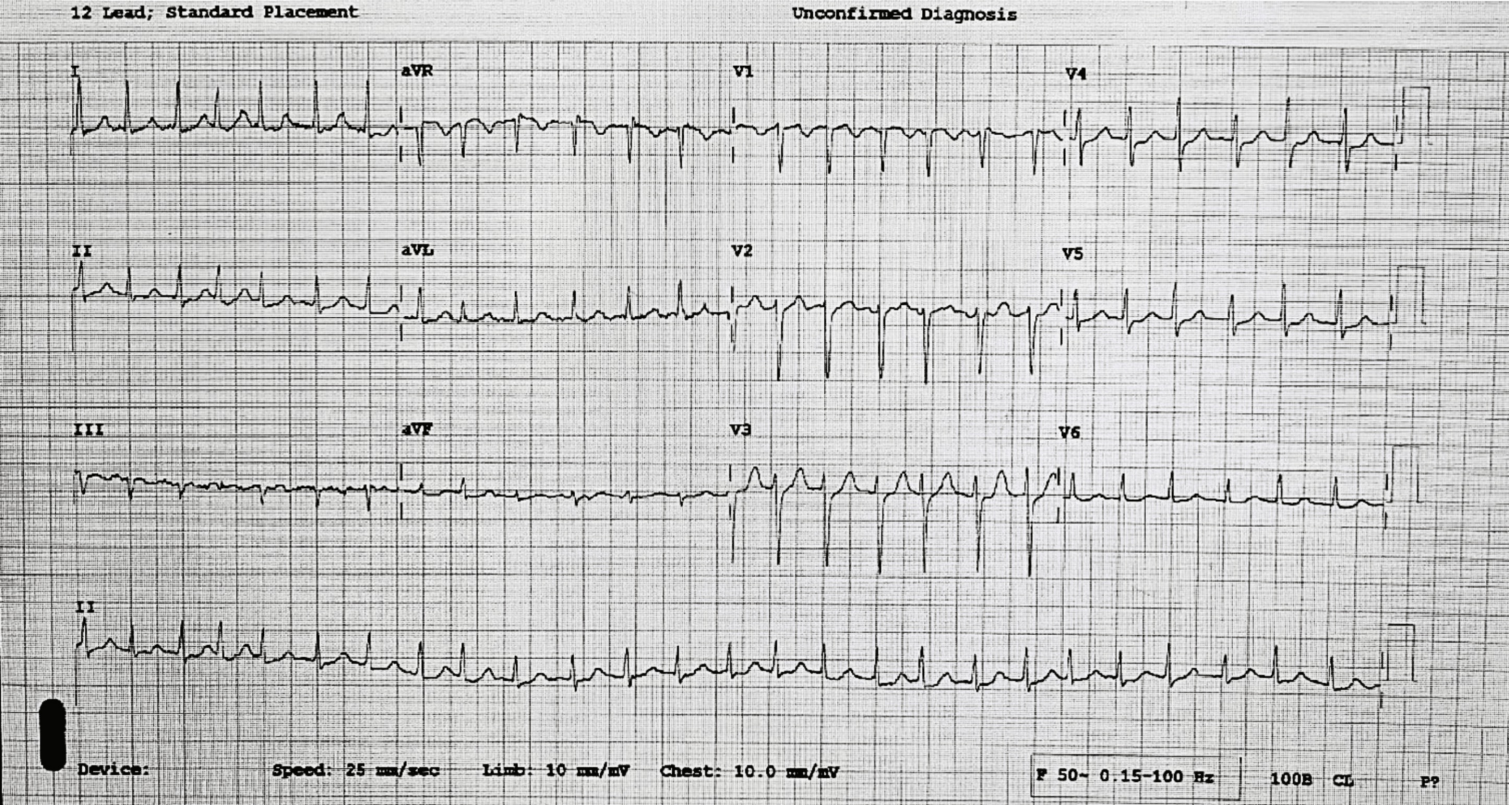
ECG demonstrating atrial fibrillation (AF) with rapid ventricular response (RVR)

On examination, the patient was tachycardic at a rate of 150-170 bpm with a blood pressure of 115/75mmHg and SpO2 of 98% on room air. An electrocardiogram (ECG) (Figure 1) confirmed AF with rapid ventricular response (RVR) without apparent ST-segment or T-wave changes. Cardio-respiratory examination was normal. An obstetric abdominal examination confirmed a non-tender gravid abdomen. Blood gas sampling revealed a pH of 7.44, glucose of 5.9 mmol/l, hemoglobin of 13.8 g/dl, and a lactate of 1.5 mmol/L. Repeat ECG demonstrated continuous fast atrial fibrillation. Focus cardiac ultrasound assessment was non-contributory due to a persistently elevated heart rate.

Our patient was diagnosed with pregnancy-related AF with RVR. It was postulated that the episode may have been precipitated by the cumulative stress of pregnancy. In the setting of pregnancy, pulmonary artery embolism was considered as a potential precipitant. However, it was considered less likely given the patient’s current heart rate and history of paroxysmal AF. In the acute management of this patient, the benefits and risks of pharmacological rate control versus rhythm control were considered. Since this patient presented with symptomatic AF with an onset of less than 12 hours, DCCV was considered [12]. Fetal medicine specialists were consulted, and it was decided, based on the benefits versus risks and the currently available literature, that DCCV with midazolam and morphine for sedation was in the best interests of the patient. 

An emergency medicine pre-procedural sedation assessment was done, and other than the inherent sedation risk in the context of pregnancy at 22 weeks, no additional sedation-related concerns were identified. Senior emergency physicians adept in managing airway and anesthetic support were considered. Under continuous monitoring in the acute resuscitation area, the patient received 5mg of Morphine and a total of 7mg of midazolam to achieve an appropriate level of sedation. The patient received a single synchronized shock at 200 joules. Post-DCCV rhythm strip confirmed sinus rhythm at a rate of 75 bpm. The patient was commenced on therapeutic enoxaparin 1mg/kg twice daily and transferred to the acute medical admission unit for monitoring and investigation. 

Blood analysis demonstrated a raised troponin of 450ng/L (reference range<16ng/L), mildly elevated pro-B-type natriuretic peptide of 474ng/l (reference range<300ng/L), normal full blood count, urea and electrolytes, thyroid function tests, and magnesium level. Computed tomography pulmonary angiography (CTPA) excluded an acute pulmonary embolism. The patient was reviewed by cardiology and maternal medicine specialists with a plan to continue six-hourly serial data monitoring and therapeutic enoxaparin and to commence aspirin 75mg daily and bisoprolol 2.5mg daily. Repeat troponin showed a down-trending level of 379ng/L. 

Following an uneventful stay, the patient was discharged home on day 3 of her admission. A transthoracic echo (TTE) demonstrated normal left ventricular systolic function for pregnancy and no significant valvular abnormalities. She remained on therapeutic enoxaparin for the next six weeks and will remain on bisoprolol and aspirin for the duration of her pregnancy. She went on to deliver a healthy baby at full term without complications.

## Discussion

According to the 2018 European Society of Cardiology (ESC) guidelines, the incidence of AF during pregnancy is approximately 27 in every 100,000 [9]. A California-based study reported an AF incidence of approximately 59.3 per 100,000 pregnancies [13]. Despite limited data on the frequency of AF in pregnancy, it is widely accepted that presentations are rapidly increasing, driven by factors such as advancing maternal age, increased parity, and cardiovascular risk factors such as obesity and metabolic syndrome. The increased cardiovascular demands of pregnancy can often unmask underlying structural or valvular cardiac disease and can exacerbate cardiomyopathies and thyroid disorders [14]. Underlying coronary artery disease is also an important risk factor and often coexists with atrial fibrillation, which is frequently missed unless investigated thoroughly with angiography [15,16].

Pregnant patients presenting with palpitations, presyncope, or syncope warrant urgent investigation [1]. AF can be promptly diagnosed through clinical examination and electrocardiogram (ECG). Given that pregnancy is a hypercoagulable state, AF management should focus on restoring normal sinus rhythm to reduce thromboembolic risk.

A 2023 multicentre study across maternity centers in the United Kingdom (UK) and Ireland highlighted the safety of DCCV in pregnancy [17]. This study was prompted by the 2014/2016 and 2015/2017 confidential inquiry (Mothers and Babies: Reducing Risk through Audit and Confidential Enquiries; (MBRRACE)-UK)), which reported that one-third of maternal cardiovascular deaths were presumed to be due to an underlying arrhythmia, with two deaths resulting from delays in delivering appropriate DCCV [10,11]. In the 2023 study, 27 women underwent a total of 29 DCCVs in pregnancy, with no maternal deaths reported and all women delivering live infants at a median gestation of 35 weeks. At first DCCV, 44% of women were in atrial fibrillation, while 30% were in atrial flutter. Most (70%) presented to an emergency department, while 30% were seen in maternity settings. Both general anesthesia and sedation were used for the cardioversion, with careful consideration given to the risk of aspiration in advanced gestations. Although fetal bradycardia occurred on two occasions post-DCCV, it was attributed to maternal hemodynamic compromise, reinforcing the need for prompt maternal stabilization [17].

Current guidelines from the European Society of Cardiology (ESC), British Cardiovascular Society (BCS), and the American Heart Association (AHA) were reviewed with respect to the management of AF in pregnancy. Management of acute AF comprises three aspects of therapy: rhythm control, rate control, and anticoagulation for thromboembolic prevention. According to the 2020 ESC guidelines, rhythm control is the preferred strategy during pregnancy. All three international guidelines suggest that direct current cardioversion (DCCV) is safe in all stages of pregnancy [1,5,13]. Immediate DCCV preceded by anticoagulation is recommended in case of hemodynamic instability, pre-excited AF, or instances where there is considerable risk to the mother or fetus. (Class 1C evidence)[5]. The BCS favors electrical cardioversion over chemical cardioversion due to the decrease in fetal risk. This is significant as pharmacological agents such as flecainide, though effective, require safety assessment, and amiodarone is generally contraindicated due to its fetotoxicity [5,6]. Electrical cardioversion presents a lower risk to both mother and fetus and the placement of defibrillator pads higher on the chest (away from the gravid uterus but not on breast tissue) minimizes the energy reaching the fetus [7]. Although amniotic fluid conducts electricity, the risk of inducing uterine contractions and preterm labor is theoretical and minimal. A comparison table outlining the potential benefits and limitations of DCCV versus pharmacological agents is included in Table 1.

**Table 1:** Potential benefits and limitations of DCCV versus other pharmacological agents. Adapted from Conti E et al. [7], ESC guidelines on the management of cardiovascular disease during pregnancy [6] and Enriquez A et al. [18]. DCCV: direct current cardioversion

	Benefit	Limitation
Antiarrhythmic medications	Flecainide or ibutilide can be considered in stable patients without structural heart disease.	Crosses placental barrier. Limited experience in pregnancy. Amiodarone is fetotoxic and only to be used if no other option is available.
Beta-blocker	Relatively safe in pregnancy	Small risk of intrauterine growth restriction, preterm birth, neonatal hypoglycemia, bradycardia and hypotension. Atenolol is contraindicated due to the risk of congenital malformation.
Calcium channel blocker	Verapamil is relatively safe during pregnancy.	Associated risks of maternal hypotension, fetal bradycardia, and tocolysis. Diltiazem should be avoided due to the risk of fetal growth restriction.
Cardiac glycoside i.e. digoxin	Relatively safe in pregnancy	Require serum level monitoring due to the risk of toxicity. The associated risk of low birth weight
Direct current cardioversion	Safe in all stages of pregnancy. No compromise to foetal blood flow. Reasonable option for arrhythmia associated with haemodynamic instability Reasonable option for refractory arrhythmia.	Minimal risk of foetal arrhythmia Theoretical risk of initiating preterm labour in later stages of pregnancy

Another report from the Netherlands showed a 93.2% success rate for DCCV during pregnancy in 41 women. The two maternal deaths reported were related to the severity of underlying heart disease, with no direct correlation to the cardioversion assumed. No additional fetal concerns were observed [3]. 

Our case reflects the complexity of managing acute AF in pregnancy in the emergency department. The recommendation to have facilities available for fetal monitoring and be cognisant of the need for emergency cesarean section proved to be a clinical challenge, especially in a facility that lacks the monitoring equipment required and might be unfamiliar with obstetric-related presentations. Through the involvement of a multidisciplinary team, including emergency physicians, cardiologists, and maternal-fetal specialists, an evidence-based decision was promptly made for the benefit of the patient and fetus. 

## Conclusions

This case highlights the complexities of managing acute AF with rapid ventricular response in pregnancy, especially in a non-obstetric emergency setting. The successful use of DCCV illustrates its safety and effectiveness, reaffirming evidence from recent studies and current international guidelines. While pregnancy-specific considerations, such as fetal monitoring and careful sedation, pose clinical challenges, this case underscores the importance of a multidisciplinary approach involving emergency physicians, cardiologists, and maternal-fetal specialists. The timely intervention resulted in a favorable outcome for both mother and baby, emphasizing that with the appropriate collaboration and adherence to evidence-based practice, AF in pregnancy can be safely managed. This case also adds to the growing body of literature supporting the role of DCCV as a primary rhythm control strategy in pregnancy, reinforcing the importance of early diagnosis and prompt management in mitigating thromboembolic risk and improving patient outcomes.

## References

[1] Miyazawa A (2024). Arrhythmias in Pregnancy. BCS.

[2] Ramlakhan KP, Kauling RM, Schenkelaars N (2022). Supraventricular arrhythmia in pregnancy. Heart.

[3] Tromp CH, Nanne AC, Pernet PJ, Tukkie R, Bolte AC (2011). Electrical cardioversion during pregnancy: safe or not?. Neth Heart J.

[4] Cauldwell M, Adamson D, Bhatia K (2023). Direct current cardioversion in pregnancy: a multicentre study. BJOG.

[5] (2021). Corrigendum to: 2020 ESC Guidelines for the diagnosis and management of atrial fibrillation developed in collaboration with the European Association of Cardio-Thoracic Surgery (EACTS). Eur Heart J.

[6] Regitz-Zagrosek V, Blomstrom Lundqvist C, Borghi C (2011). ESC Guidelines on the management of cardiovascular diseases during pregnancy: the Task Force on the Management of Cardiovascular Diseases during Pregnancy of the European Society of Cardiology (ESC). Eur Heart J.

[7] Conti E, Cascio ND, Paluan P (2024). Pregnancy arrhythmias: Management in the emergency department and critical care. J Clin Med.

[8] Tamirisa KP, Elkayam U, Briller JE (2022). Arrhythmias in pregnancy. JACC Clin Electrophysiol.

[9] Regitz-Zagrosek V, Roos-Hesselink JW, Bauersachs J (2019). 2018 ESC Guidelines for the management of cardiovascular diseases during pregnancy. Kardiol Pol.

[10] Knight M (2019). The findings of the MBRRACE-UK confidential enquiry into maternal deaths and morbidity. Obs Gyn Reprod Med.

[11] Draper ES, Gallimore ID, Smith LK (2022). MBRRACE-UK Perinatal Mortality Surveillance Report: UK Perinatal Deaths for Births from January to December. http://www.npeu.ox.ac.uk/assets/downloads/mbrrace-uk/reports/perinatal-surveillance-report-2020/MBRRACE-UK_Perinatal_Surveillance_Report_Technical_document.pdf.

[12] Camm AJ, Kirchhof P, Lip GY (2010). Guidelines for the management of atrial fibrillation: the task force for the management of atrial fibrillation of the European Society of Cardiology (ESC). Eur Heart J.

[13] Lee MS, Chen W, Zhang Z (2016). Atrial fibrillation and atrial flutter in pregnant women-a population-based study. J Am Heart Assoc.

[14] Sliwa K, Hilfiker-Kleiner D, Petrie MC (2010). Current state of knowledge on aetiology, diagnosis, management, and therapy of peripartum cardiomyopathy: a position statement from the Heart Failure Association of the European Society of Cardiology Working Group on peripartum cardiomyopathy. Eur J Heart Fail.

[15] Sharma YP, Batta A, Makkar K (2022). Angiographic profile and outcomes in persistent non-valvular atrial fibrillation: A study from tertiary care center in North India. Indian Heart J.

[16] Mekhael M, Marrouche N, Hajjar AH, Donnellan E (2024). The relationship between atrial fibrillation and coronary artery disease: Understanding common denominators. Trends Cardiovasc Med.

[17] Youssef G (2024). Management of atrial fibrillation during pregnancy. E J Cardio Pract (Euro).

[18] Enriquez AD, Economy KE, Tedrow UB (2014). Contemporary management of arrhythmias during pregnancy. Circ Arrhythm Electrophysiol.

